# Prediction of clear cell renal cell carcinoma ≤ 4cm: visual assessment of ultrasound characteristics versus ultrasonographic radiomics analysis

**DOI:** 10.3389/fonc.2024.1298710

**Published:** 2024-07-23

**Authors:** Fan Yang, Dai Zhang, Li-Hui Zhao, Yi-Ran Mao, Jie Mu, Hai-Ling Wang, Liang Pang, Shi-Qiang Yang, Xi Wei, Chun-Wei Liu

**Affiliations:** ^1^ Department of Ultrasound Diagnosis and Treatment, Tianjin Medical University Cancer Institute and Hospital, National Clinical Research Center for Cancer, Tianjin, China; ^2^ Key Laboratory of Cancer Prevention and Therapy, Tianjin’s Clinical Research Center for Cancer, Tianjin Medical University, Tianjin, China; ^3^ Department of Urology, Tianjin Occupational Diseases Precaution and Therapeutic Hospital, Tianjin, China; ^4^ Department of Urology, Tianjin First Central Hospital, Tianjin, China; ^5^ Department of Cardiology, Tianjin Chest Hospital, Tianjin University, Tianjin, China

**Keywords:** small renal tumor, clear cell renal cell carcinoma, ultrasound, radiomics, renal angiomyolipoma

## Abstract

**Objective:**

To investigate the diagnostic efficacy of the clinical ultrasound imaging model, ultrasonographic radiomics model, and comprehensive model based on ultrasonographic radiomics for the differentiation of small clear cell Renal Cell Carcinoma (ccRCC) and Renal Angiomyolipoma (RAML).

**Methods:**

The clinical, ultrasound, and contrast-enhanced CT(CECT) imaging data of 302 small renal tumors (maximum diameter ≤ 4cm) patients in Tianjin Medical University Cancer Institute and Hospital from June 2018 to June 2022 were retrospectively analyzed, with 182 patients of ccRCC and 120 patients of RAML. The ultrasound images of the largest diameter of renal tumors were manually segmented by ITK-SNAP software, and Pyradiomics (v3.0.1) module in Python 3.8.7 was applied to extract ultrasonographic radiomics features from ROI segmented images. The patients were randomly divided into training and internal validation cohorts in the ratio of 7:3. The Random Forest algorithm of the Sklearn module was applied to construct the clinical ultrasound imaging model, ultrasonographic radiomics model, and comprehensive model. The efficacy of the prediction models was verified in an independent external validation cohort consisting of 69 patients, from 230 small renal tumor patients in two different institutions. The Delong test compared the predictive ability of three models and CECT. Calibration Curve and clinical Decision Curve Analysis were applied to evaluate the model and determine the net benefit to patients.

**Results:**

491 ultrasonographic radiomics features were extracted from 302 small renal tumor patients, and 9 ultrasonographic radiomics features were finally retained for modeling after regression and dimensionality reduction. In the internal validation cohort, the area under the curve (AUC), sensitivity, specificity, and accuracy of the clinical ultrasound imaging model, ultrasonographic radiomics model, comprehensive model, and CECT were 0.75, 76.7%, 60.0%, 70.0%; 0.80, 85.6%, 61.7%, 76.0%; 0.88, 90.6%, 76.7%, 85.0% and 0.90, 92.6%, 88.9%, 91.1%, respectively. In the external validation cohort, AUC, sensitivity, specificity, and accuracy of the three models and CECT were 0.73, 67.5%, 69.1%, 68.3%; 0.89, 86.7%, 80.0%, 83.5%; 0.90, 85.0%, 85.5%, 85.2% and 0.91, 94.6%, 88.3%, 91.3%, respectively. The DeLong test showed no significant difference between the clinical ultrasound imaging model and the ultrasonographic radiomics model (Z=-1.287, P=0.198). The comprehensive model showed superior diagnostic performance than the ultrasonographic radiomics model (Z=4. 394, P<0.001) and the clinical ultrasound imaging model (Z=4. 732, P<0.001). Moreover, there was no significant difference in AUC between the comprehensive model and CECT (Z=-0.252, P=0.801). Both in the internal and external validation cohort, the Calibration Curve and Decision Curve Analysis showed a better performance of the comprehensive model.

**Conclusion:**

It is feasible to construct an ultrasonographic radiomics model for distinguishing small ccRCC and RAML based on ultrasound images, and the diagnostic performance of the comprehensive model is superior to the clinical ultrasound imaging model and ultrasonographic radiomics model, similar to that of CECT.

## Introduction

With the improvement of imaging techniques, the incidence of renal cell carcinoma (RCC) has been steadily increasing at a rate of 2%-4% every year (1), among which the proportion of patients diagnosed with small RCC (diameter ≤ 4 cm) has been constantly increasing (2). Recently, small renal tumors have become a hot topic in research. About 20-30% of small renal tumors are benign, and renal angiomyolipoma (RAML) is the most common pathology type (3). Clear cell renal cell carcinoma (ccRCC) is the most common pathology type of RCC. There’s a lack of typical malignant ultrasound features (necrosis or tumor embolism) in small renal tumors less than 4cm, which makes it difficult to discriminate small ccRCC from RAML. The low-fat content results in hypoechoicity on ultrasound in fat-poor renal angiomyolipoma (fpRAML), similar to small renal carcinomas. Transabdominal ultrasonography is a common method in preoperative imaging examination of renal tumors, but the small renal tumors may be ignored due to the above sonographic characteristics.

In clinical practice, the identification of ccRCC and RAML is mainly based on contrast-enhanced CT (CECT). However, CECT is an invasive procedure involving intravenous contrast injection, making it unsuitable for patients with renal dysfunction or iodine contrast allergies. Ultrasonography examination is widely used in clinical practice and is inexpensive, feasible, and reproducible. Moreover, the small ccRCC may be untypical on CECT, and their presentation could be easily confused with RAML, especially low-fat RAML (4). In case of an unclear diagnosis, a repeated examination is required during the follow-up period. So, repeated ultrasound examinations may be more acceptable to these patients. Improvement of the ultrasound diagnostic capability in clinical physical examination will be beneficial.

With the development of artificial intelligence, radiomics prediction models have gained attention in cancer diagnosis (5, 6). Radiomics can extract inaccessible feature data from medical images with high throughput and has great application prospects in predicting the biological behavior of tumors (7, 8). In recent years, few studies have been reported on ultrasonographic radiomics to identify small ccRCC. It is unclear whether the diagnostic performance could be improved using ultrasonographic radiomics in these patients. In this current study, we investigated the feasibility of ultrasonographic radiomics to discriminate ccRCC and RAML by constructing a clinical ultrasound imaging model, ultrasonographic radiomics model, and comprehensive model. We also compared the diagnostic efficacy between the above models and CECT.

## Materials and methods

### Study population

**Figure 1:**
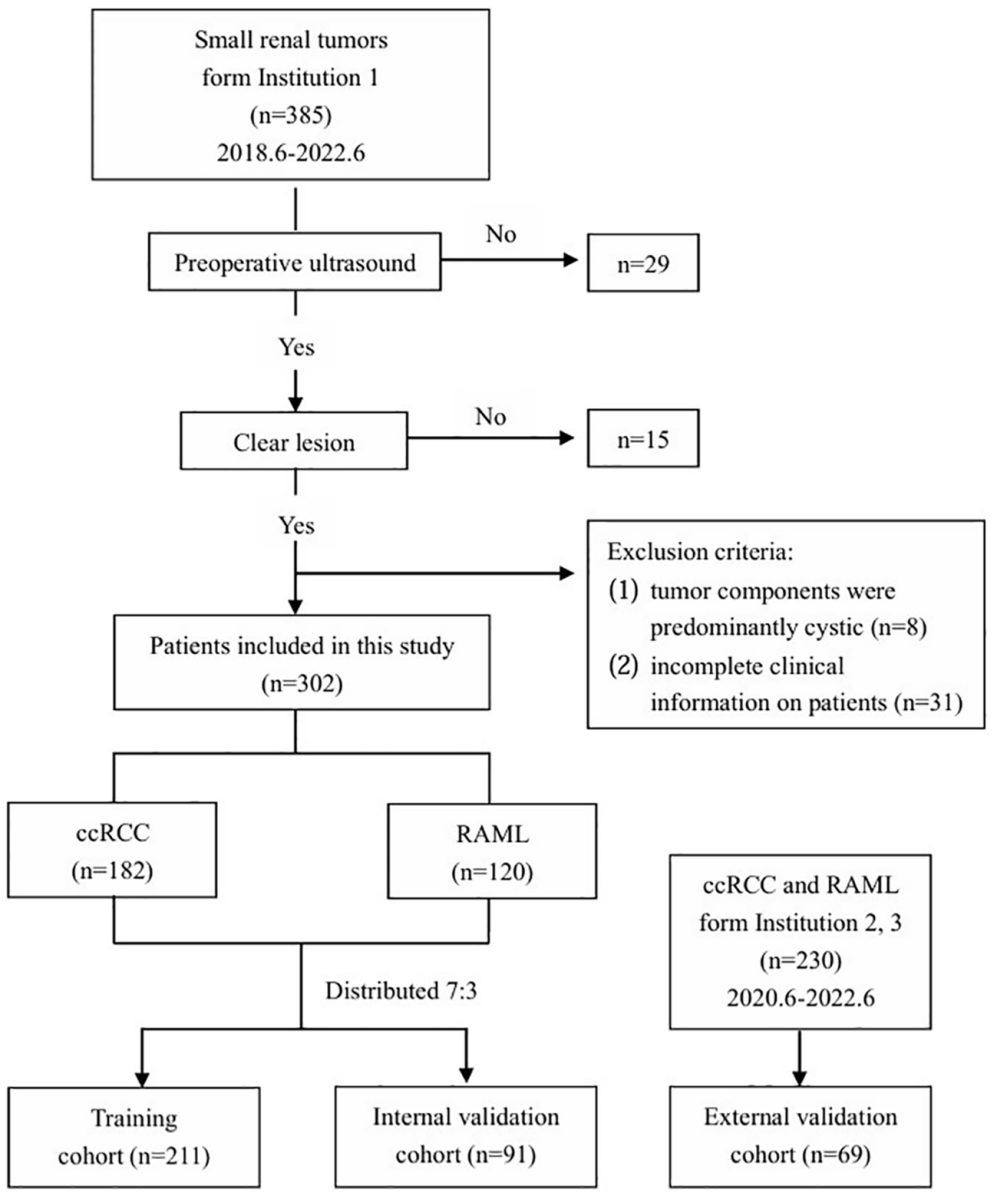
Flowchart of inclusion and exclusion of the study population.

This retrospective study was approved by the ethics committee of Tianjin Medical University Cancer Institute and Hospital (bc2023079). From June 2018 to June 2022, 385 small renal tumor patients with a histological examination at Tianjin Medical University Cancer Institute (institution 1) were retrospectively recruited to construct training and internal validation cohorts of the model. Another 230 small renal tumor patients with a histological examination from Tianjin First Central Hospital, and Tianjin Occupational Diseases Precaution and Therapeutic Hospital (institutions 2 and 3) were retrospectively recruited, constituting an independent external validation cohort to verify the efficacy of the prediction model. The inclusion criteria were as follows: (1) patients performed an ultrasonic examination and CECT within 2 weeks before the operation, and the images of the tumor’s largest diameter were clear, (2) the diagnosis of ccRCC or RAML was confirmed by postoperative pathology, (3) patients had no previous history of other malignancies, (4) maximum diameter of renal tumor ≤ 4cm. The excluding standards were as follows: (1) there were significant artifacts in the ultrasound or CT images, (2) tumor components were predominantly cystic (the solid component was less than 25%) (9), and (3) incomplete clinical information on patients. As a result, 302 patients with 302 small renal tumors were finally enrolled in our study to construct ultrasonographic radiomics models and internal validation; and 69 patients for external validation (**Figure 1**). Patients in institution 1 were divided into the ccRCC group (n = 182; 107 men and 75 women; mean age 56.85 ± 10.71 years) and RAML group (n = 120; 57 men and 63 women; mean age 53.64 ± 12.23 years). The mean age of the external validation cohort was 55.72 ± 14.58 years (38 men and 31 women).

### Ultrasonography and CT scanning methods and image analysis

Color Doppler ultrasonic diagnostic apparatus of PHILIPS EPIQ5, Toshiba Aplio 500, and 800 were used. Transabdominal ultrasound was performed using a convex array probe with 1~6MHz. Patients should be fasting for 8-12 hours to show the largest section of tumors clearly. We performed a multisectional examination of both kidneys in the supine, lateral, or prone position. Ultrasonographic features of renal tumors were recorded, including tumor location, maximum diameter, tumor boundaries, echo pattern, presence of calcifications, necrotic cystic degeneration, and blood flow signals.

Preoperative CECT was performed on multiple scanners: Siemens Somatom Definition, GE HiSpeed 16, and Philips Brilliance 64. Acquisition parameters were as follows: tube voltage, 120–140 kV; automated varied milliampere-second settings; collimation width, 1.5 mm. CT and CECT features included fat density assessment, peak enhancement degree, homogeneity of enhancement, and the velocity of contrast in and out.

Ultrasonographic and CECT imaging were independently assessed by two sonographers and two radiologists (all with more than 10 years of experience). They were blinded to the pathology results. When the diagnostic results were inconsistent, they reached a consensus through discussion. Clinical information of these patients was recorded, including sex, age, and clinical symptoms.

### Segmentation and pre-processing of ultrasound images

**Figure 2:**
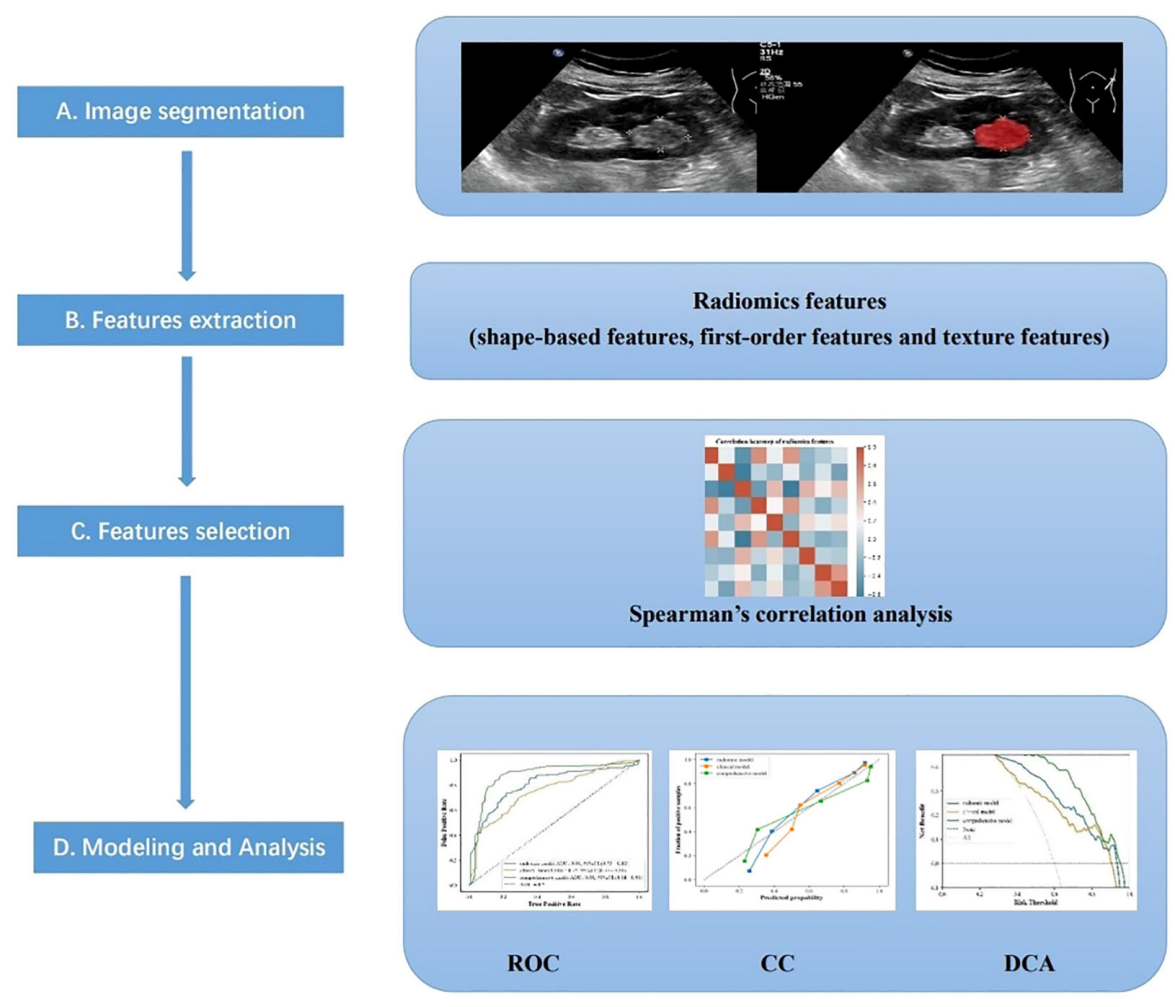
The main procedure performed in this study comprised four steps: **(A)** ultrasound imaging and tumor segmentation, **(B)** image processing and feature extraction, **(C)** feature selection, and **(D)** modeling, and Analysis.

**Figure 3:**
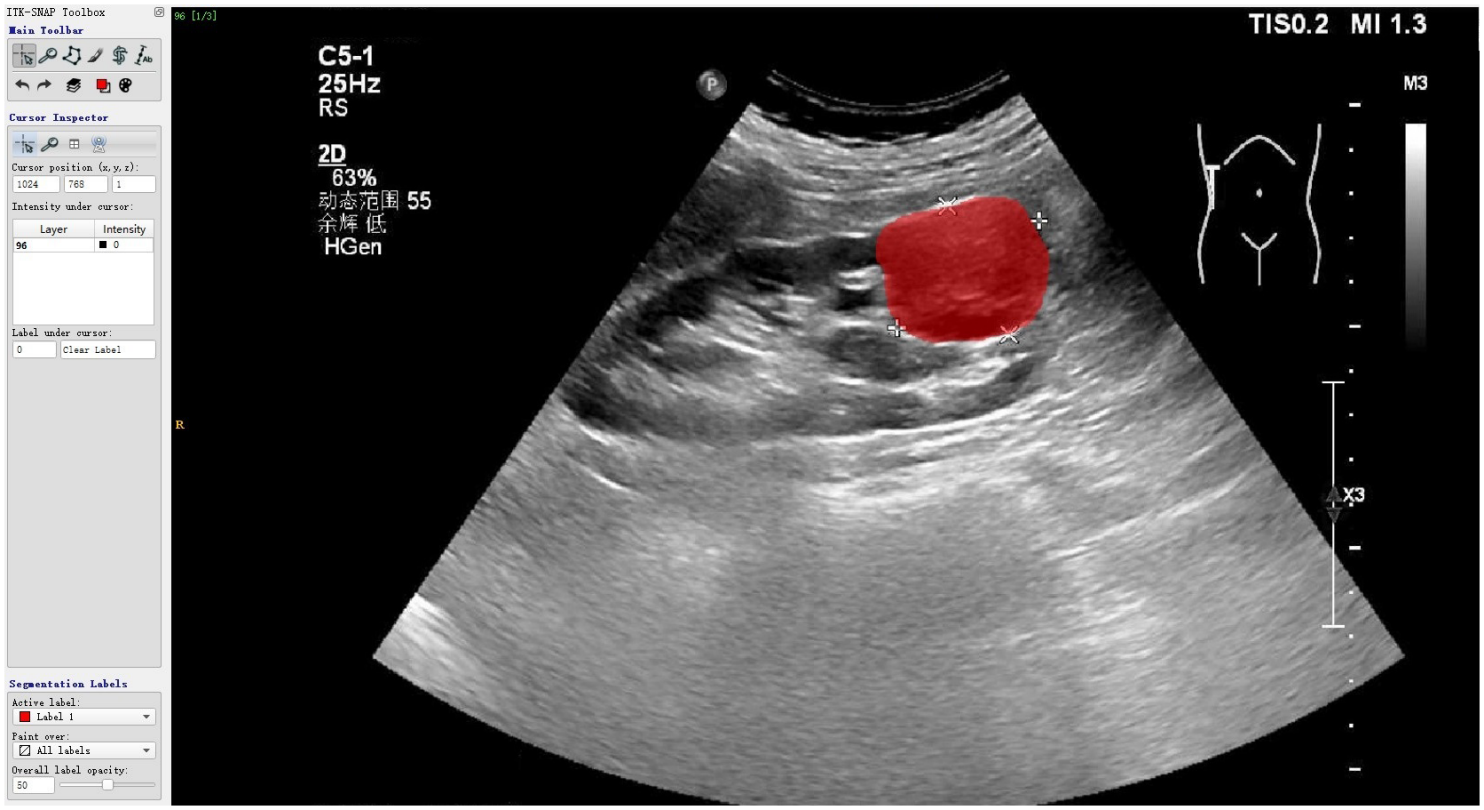
The renal tumor edge was outlined as the region of interest by ITK-SNAP software.

The framework of this study is illustrated in **Figure 2**. Ultrasound images of the largest renal tumor cross-section were imported into ITK-SNAP software (version v 3.8.0, www.itksnap.org), and the tumor edges were manually outlined as the region of interest (ROI) by sonographer A with more than 10 years’ experience (**Figure 3**). Two weeks later, sonographer A and sonographer B (with 5 years of experience) made ROI outlining from 50 renal tumor images randomly, to assess intra- and inter-observer correlation coefficients (ICCs).

### Radiomics features extraction

Z-Score was performed to standardize the data of different orders before radiomics feature extraction. The Pyradiomics module of Python 3.8.7 (v3.0.1) was used to extract the radiomics features of ROIs, including shape-based features, first-order features, and structural texture features. The structural texture features included a gray level cooccurrence matrix (GLCM), gray level run-length matrix (GLRLM), gray level size zone matrix (GLSZM), and gray level dependence matrix (GLDM). ICCs were used to evaluate the agreement of feature extraction. The intra-observer ICC was calculated based on two feature extractions by sonographer A, and inter-observer ICC was calculated based on the features extracted firstly by sonographer A and subsequently by sonographer B. Features with better consistency (ICC > 0.9) were retained. The maximum relevance-minimum redundancy features were obtained by max-relevance and min-redundancy (MRMR) algorithm filtering. The covariance between ultrasound features was assessed by calculating the Variance Inflation Factor (VIF). Finally, the high-stability radiomics features were subjected to Spearman’s correlation analysis, with a correlation coefficient threshold of 0.7.

### Construction of ultrasonographic radiomics model

The patients were randomly divided into training and internal validation cohorts in the ratio of 7:3. The Random forest algorithm (RFA) of the Sklearn module (Python 3.8.7) was applied to construct the clinical ultrasound imaging model, ultrasonographic radiomics model, and comprehensive model, to predict diagnostic efficacy for small ccRCCs. Both feature extraction and model construction used ten-fold cross-validation and parameter tuning to optimize the predictive performance.

### Statistical analysis

All data were analyzed using the SPSS Statistics software version 23.0 (IBM, Armonk, NY, USA), Python3.8.7 and R software version 4.2.2. All categorical variables were expressed as numbers(n) and percentages, and continuous variables were expressed as mean value ± standard deviation (SD) or median ± inter-quartile range (IQR). χ² test was used to compare the clinical ultrasound characteristics between patients in ccRCC and RAML groups. The diagnostic ability of the ultrasound imaging model, ultrasonographic radiomics model, comprehensive model, and CECT for ccRCC were assessed by the receiver operating characteristic (ROC) curve, and the area under the curve (AUC), sensitivity, specificity, and accuracy of three models and CECT were calculated separately. The AUC values of different models and CECT were compared by the Delong test. The performance of the three models was evaluated by the Calibration Curve. Clinical Decision Curve Analysis was also applied to determine the net benefit of patients. P<0.05 was considered statistically significant.

## Results

### Comparison of clinical ultrasound and CECT characteristics

**Figure 4:**
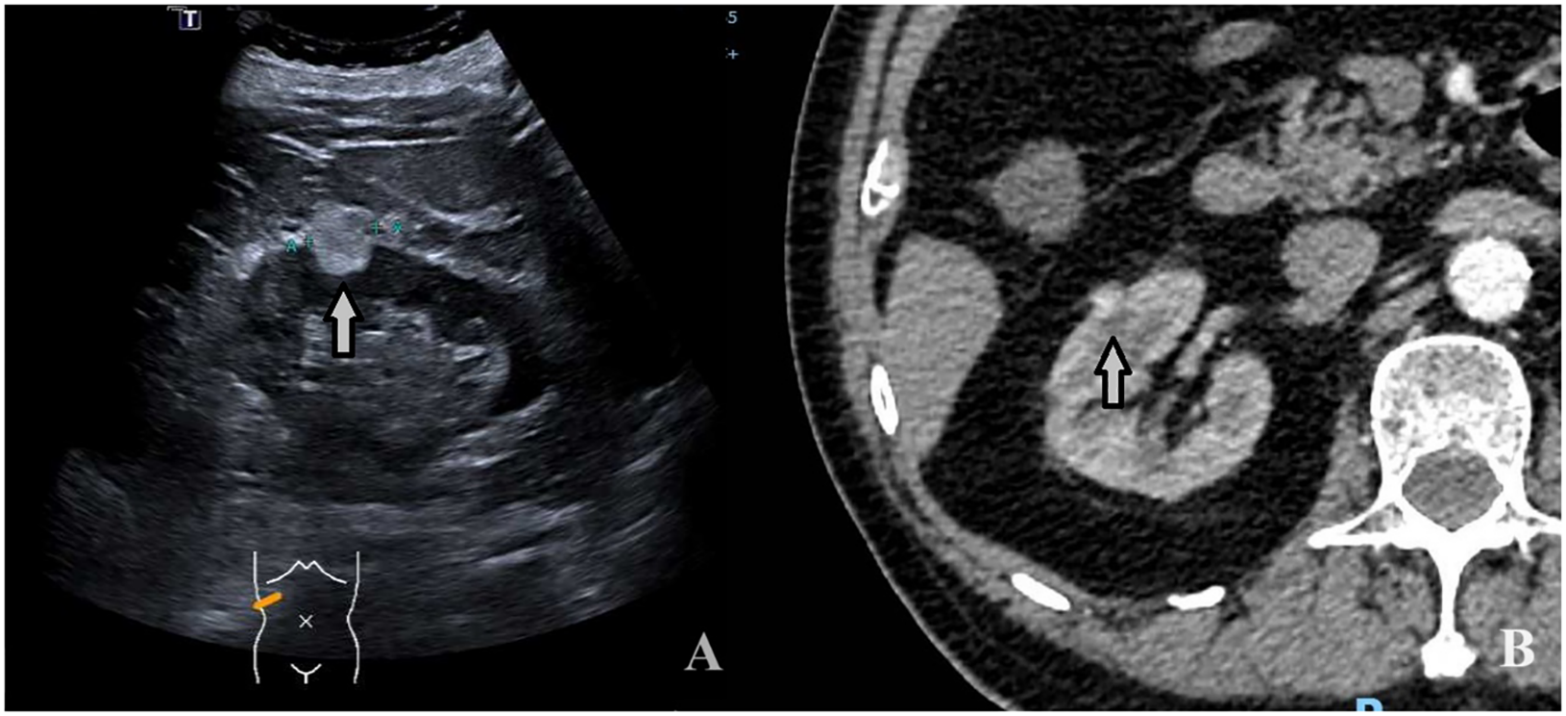
A 74-year-old man with a 1.9 × 1.4cm RAML. **(A)** Ultrasound demonstrated a mildly hyperechoic mass located in the middle pole of the right kidney (arrow). **(B)** CECT: the mass showed inhomogeneous hyperenhancement (arrow).

**Figure 5:**
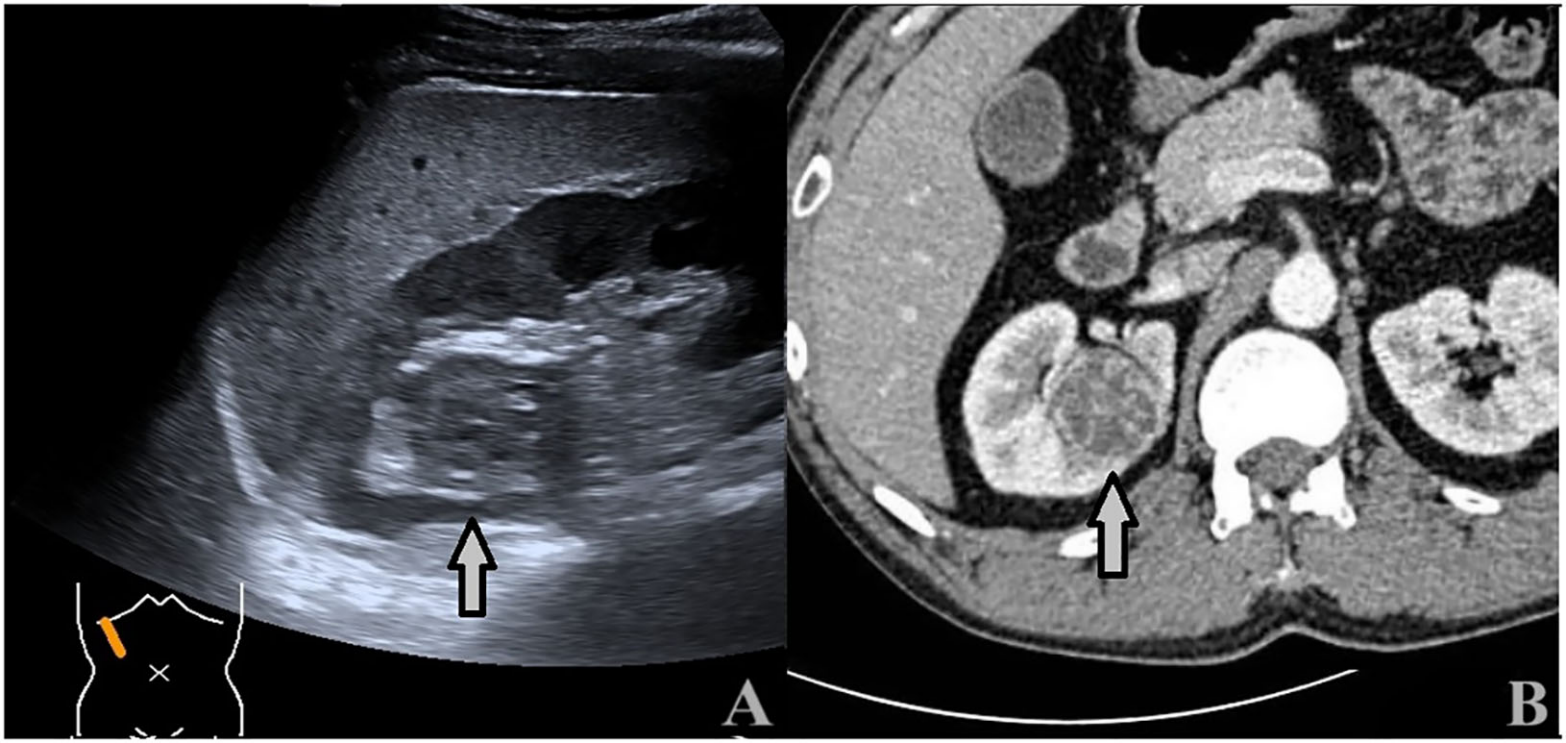
A 43-year-old man with a 3.5 × 3.4cm ccRCC. **(A)** Ultrasound showed a heterogeneous hypoechoic mass with intratumoral fluid areas located at the upper pole of the right kidney (arrows). **(B)** CECT: the tumor showed inhomogeneous hyperenhancement with many unenhanced areas (arrows).

**Figure 6:**
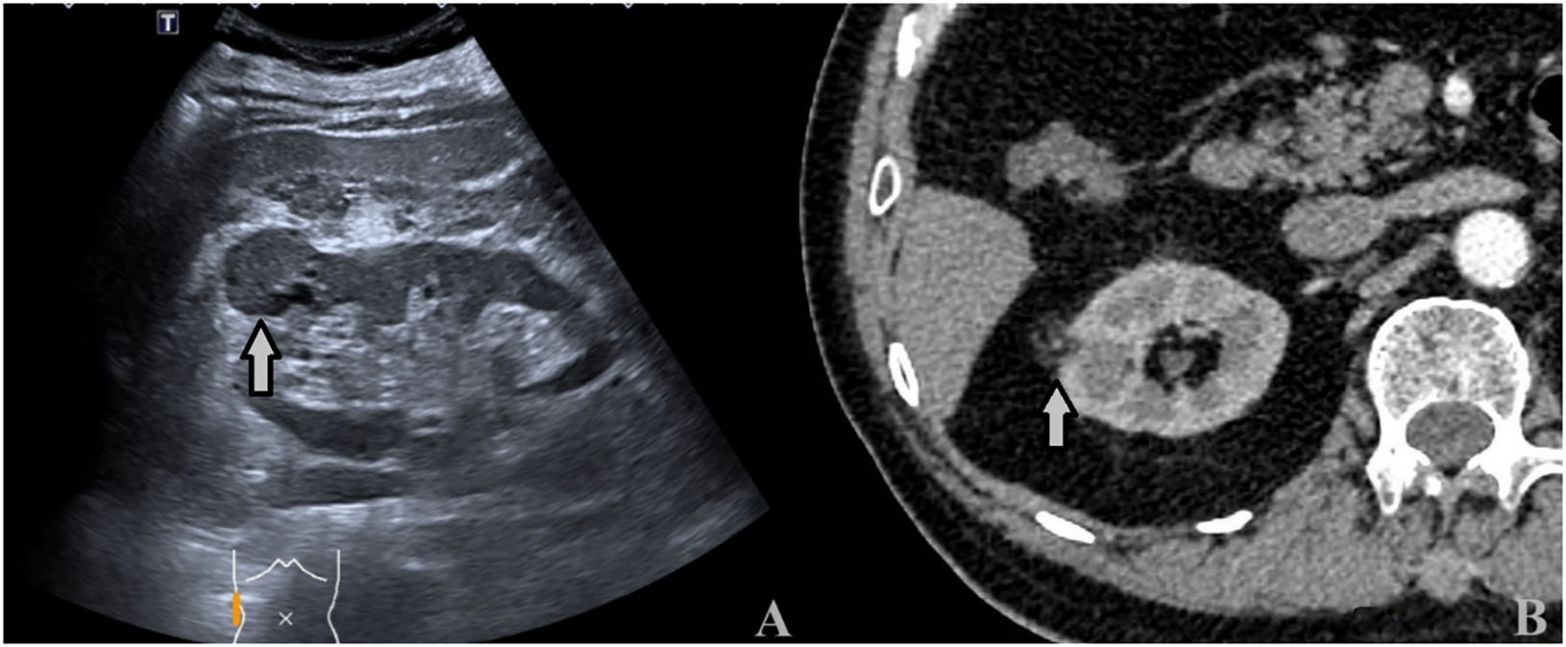
A 64-year-old man with a 3.2 × 2.8cm ccRCC. **(A)** Ultrasound showed a heterogeneous hypoechoic mass with intratumoral fluid areas located at the upper pole of the right kidney (arrows). **(B)** CECT: the tumor showed inhomogeneous hyperenhancement with many unenhanced areas (arrows).

In this study, 302 small renal tumor patients, comprising 182 with ccRCC and 120 with RAML, were enrolled as the training and internal validation cohorts. There were significant differences in the distribution of gender, clinical symptoms, echo pattern, necrotic cystic degeneration, blood flow signals, CT presence of bulk fat, and homogeneity of enhancement, but no significant differences in age, location, tumor boundaries, calcification, peak enhancement degree, existent of fast-in and fast-out between the two groups (P<0.05, **Table 1**; **Figures 4**–**6**).

**Table 1:** Clinical ultrasound and CECT characteristics of 302 small renal tumor patients.

	ccRCC (n=182)	RAML (n=120)	χ2	*P*
Gender				
Male	105 (57.7%)	57 (47.5%)	3.021	0.082
Female	77 (42.3%)	63 (52.5%)		
Age				
<50 years old	43 (23.6%)	34 (28.3%)	0.843	0.358
≥50 years old	139 (76.4%)	86 (71.7%)		
Clinical symptoms				
Hematuria	29 (15.9%)	8 (6.7%)	5.777	0.016
No hematuria	153 (84.1%)	112 (93.3%)		
Ultrasound characteristics				
Location				
Left kidney	101 (55.5%)	59 (49.2%)	1.162	0.281
Right kidney	81 (44.5%)	61 (50.8%)		
Tumor boundaries				
Clear	140 (76.9%)	95 (79.2%)	0.211	0.646
Unclear	42 (23.1%)	25 (20.8%)		
Echo pattern				
Hypoechoic	87 (47.8%)	31 (25.8%)	16.566	0.000
Isoechoic	39 (21.4%)	28 (23.3%)		
Hyperechoic	56 (30.8%)	61 (50.9%)		
Calcification				
Existent	38 (20.9%)	15 (12.5%)	3.509	0.061
Non-existent	144 (79.1%)	105 (87.5%)		
Necrotic cystic degeneration				
Existent	44 (24.2%)	9 (7.5%)	13.898	0.000
Non-existent	138 (75.8%)	111 (92.5%)		
blood flow signals				
Existent	102 (56.0%)	51 (42.5%)	5.307	0.021
Non-existent	80 (44.0%)	69 (57.5%)		
CT and CECT characteristics				
Presence of bulk fat				
Existent	73 (40.1%)	76 (63.3%)	15.604	0.000
Non-existent	109 (59.9%)	44 (36.7%)		
Peak enhancement degree				
hyper- enhancement	116 (63.7%)	87 (72.5%)	2.521	0.112
iso-/hypo-enhancement	66 (36.3%)	33 (27.5%)		
Homogeneity of enhancement				
homogeneous	78 (42.9%)	69 (57.5%)	6.207	0.013
inhomogeneous	104 (57.1%)	51 (42.5%)		
Fast-in and fast-out				
Existent	103 (56.6%)	62 (51.7%)	0.708	0.400
Non-existent	79 (43.4%)	58 (48.3%)		

P-values indicate comparisons between ccRCC and RAML groups.

ccRCC, clear cell Renal Cell Carcinoma; RAML, Renal Angiomyolipoma; CECT, contrast-enhanced CT.

### Selection of ultrasonographic radiomics features

**Figure 7:**
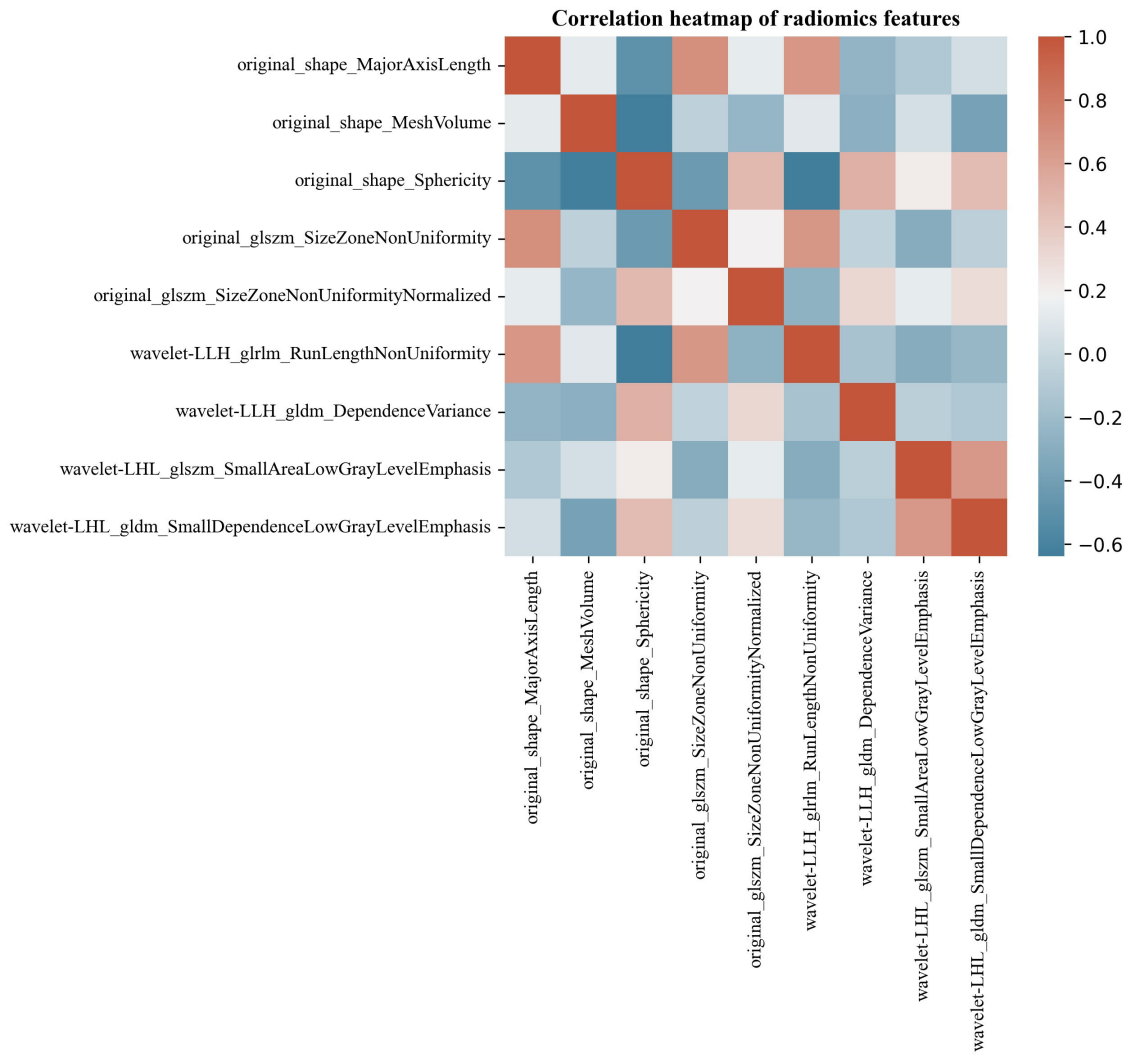
Spearman correlation heatmap of renal tumor ultrasonographic radiomics features. The color indicates a correlation: the darker the color, the higher the correlation (red indicates a positive correlation, and blue indicates a negative correlation).

The Pyradiomics software package extracted 491 ultrasonographic radiomics features. 9 ultrasonographic radiomics features were finally retained after regression dimensionality reduction processing, including 3 Shape, 1 GLRLM, 3 GLSZM, and 2 GLDM features. Spearman correlation heatmap of radiomics features is shown in **Figure 7**.

### Diagnostic efficacy of predictive models

**Figure 8:**
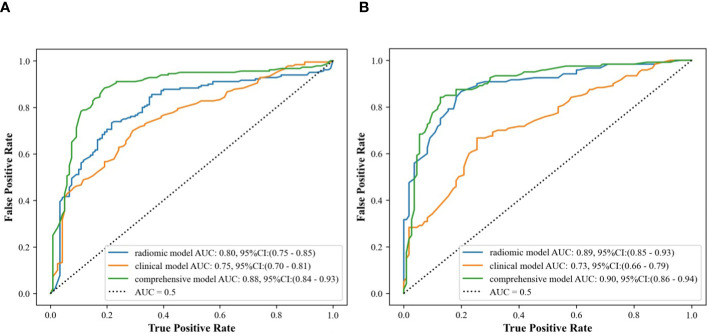
ROC curves of clinical ultrasound imaging model, ultrasonographic radiomics model,. and comprehensive model in the internal **(A)** and external **(B)** validation cohort.

**Figure 9:**
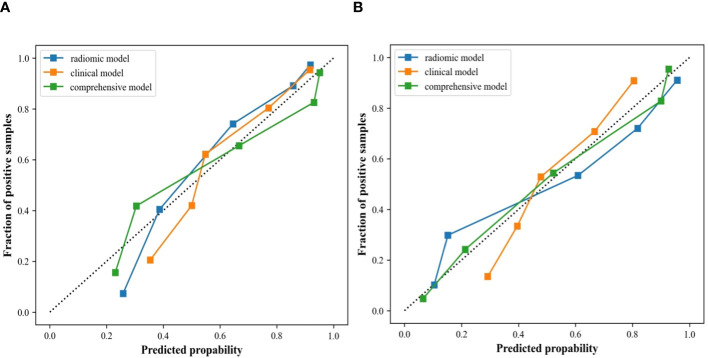
Calibration curves of the clinical ultrasound imaging model, ultrasonographic radiomics model, and comprehensive model in the internal **(A)** and external **(B)** validation cohort.

**Figure 10:**
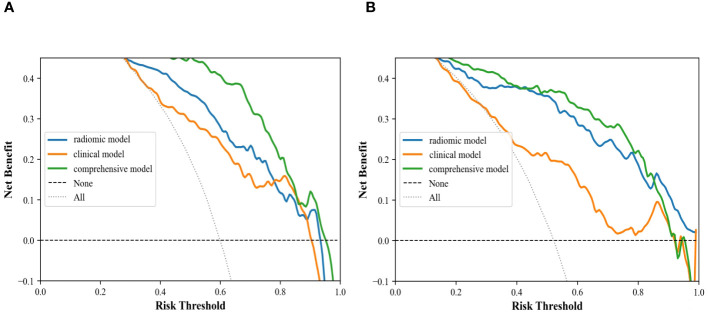
Decision curve analysis of clinical ultrasound imaging model, ultrasonographic radiomics model, and comprehensive model in the internal **(A)** and external **(B)** validation cohort.

In the internal validation cohort, AUC, sensitivity, specificity, and accuracy of the clinical ultrasound imaging model, ultrasonographic radiomics model, comprehensive model, and CECT for ccRCC diagnostic prediction were 0.75, 76.7%, 60.0%, 70.0%; 0.80, 85.6%, 61.7%, 76.0%; 0.88, 90.6%, 76.7%, 85.0% and 0.90, 92.6%, 88.9%, 91.1%, respectively. In the external validation cohort, AUC, sensitivity, specificity, and accuracy of the three models and CECT were 0.73, 67.5%, 69.1%, 68.3%; 0.89, 86.7%, 80.0%, 83.5%; 0.90, 85.0%, 85.5%, 85.2% and 0.91, 94.6%, 88.3%, 91.3%, respectively (**Figure 8**; **Table 2**). In the internal validation cohort, the DeLong test demonstrated no significant difference in AUC between the clinical ultrasound imaging model and ultrasonographic radiomics model (Z=-1.287, P=0.198), whereas the comprehensive model was superior to the ultrasonographic radiomics model (Z=4. 394, P<0.001) and clinical ultrasound imaging model (Z=4. 732, P<0.001). Moreover, there was no significant difference in AUC between the comprehensive model and CECT (Z=-0.252, P=0.801). The Calibration curve indicated a better performance of the comprehensive model (**Figure 9**), while Decision Curve Analysis showed a superior clinical utility of the comprehensive model (**Figure 10**).

**Table 2:** Comparison of the predictive efficacy of clinical ultrasound imaging model, ultrasonographic radiomics model, comprehensive model, and CECT in the internal and external validation cohorts.

	Model	AUC (95%CI)	Sensitivity	Specificity	Accuracy
Internal validation cohort (n=90)	Clinical ultrasound imaging model	0.75 (0.70-0.81)	76.7%	60.0%	70.0%
	Ultrasonographic radiomics model	0.80 (0.75-0.85)	85.6%	61.7%	76.0%
	Comprehensive model	0.88 (0.84-0.93)	90.6%	76.7%	85.0%
	CECT	0.90 (0.84-0.98)	92.6%	88.9%	91.1%
External validation cohort (n=69)	Clinical ultrasound imaging model	0.73 (0.66-0.79)	67.5%	69.1%	68.3%
	Ultrasonographic radiomics model	0.89 (0.85-0.93)	86.7%	80.0%	83.5%
	Comprehensive model	0.90 (0.86-0.94)	85.0%	85.5%	85.2%
	CECT	0.91 (0.87-0.95)	94.6%	88.3%	91.3%

## Discussion

The clinical symptoms are usually untypical in patients with small ccRCC. Many patients are discovered incidentally during radiologic examinations (10, 11). It is also more complex considering clinical decision-making (12–14), including a variety of interventions available for these patients: renal tumor biopsy, partial nephrectomy, radical nephrectomy, thermal ablation, and follow-up monitoring (6, 15, 16). Therefore, the evaluation of imaging features of renal tumors has gradually evolved from morphological presentation to criteria based on histological features and molecular typing features (17, 18). Radiomics can quantitatively assess the heterogeneity of tumors and can be applied to differentiate renal carcinoma from RAML, assess the biological behavior of tumors, and predict the risk of recurrence or survival (19–21). Several studies have reported radiomics models established by CT or MR images, demonstrating their utility in identifying benign and malignant renal tumors and predicting pathological grading (22–25). However, reports on the establishment of radiomics models of ultrasound, the most convenient imaging examination for screening renal tumors, are rather rare.

In this study, the sensitivity of the internal validation cohort of the clinical ultrasound imaging model for the prediction of ccRCC was 76.7% and the accuracy was 70.0%. Although there were statistically significant differences in gender, clinical symptoms, echo pattern, necrotic cystic degeneration, and blood flow signals between patients in the ccRCC group and RAML group, 30.0% of small renal carcinomas were still misdiagnosed in this model. The reason may be that patients with small ccRCC do not have the specificity of clinical presentation and have a complex and varied pathohistological structure. Whereas ccRCC and fpRAML may exhibit some similar ultrasound characteristics (26, 27). The comprehensive model showed superior ability in predicting ccRCC, with 91% of sensitivity and 77% of specificity. The model extracted 491 ultrasonographic radiomics features, which were processed by regression dimensionality reduction, and finally retained 9 stable ultrasonographic radiomics features. Among them, Shape features described the morphological information of renal tumors. Major axis length, Mesh volume, and Sphericity described the similarity of renal tumor morphology to the standard sphere. Renal tumors in the RAML group had a smaller long-axis length and were closer to spherical than those in the ccRCC group. GLSZM was a count of the number of groups of interconnected neighboring pixels or voxels with the same gray level form the basis for the matrix (28). GLRLM provided information about the spatial distribution of runs of consecutive pixels with the same gray level, assessing the percentage of pixels or voxels within the ROI that are part of the runs and therefore reflect graininess (29). GLDM was also a count matrix that holds information about the number of “dependent” pixels and the number of occurrences of all pixels in the image. All the above three features belonged to texture features, which suggested that the tumors in the ccRCC group had poor texture consistency and a significant effect of non-periodic or speckled texture in ultrasound images compared to those in the RAML group. These findings indicated higher tumor heterogeneity in ccRCC. Compared with the RAML group, tumors in the ccRCC group had more irregular morphology, wider image signal distribution, and rougher texture features. So, combining ultrasonographic radiomics features and clinical ultrasound imaging features, the comprehensive model showed better diagnostic efficacy. The comprehensive model improved the sensitivity and accuracy of ccRCC prediction to 90.6% and 85.0%, which was similar to previous studies (20, 30–32). Our study suggested that ultrasonographic radiomics features could compensate for the shortcomings of clinical ultrasound imaging features and improve the predictive efficacy of small ccRCC. The Calibration Curve and Decision Curve Analysis of the three models also validated that the comprehensive model had a higher net benefit and a better performance in predicting patients with small ccRCC.

Our study had several improvements compared with the previous radiomics studies. Firstly, we compared the diagnostic efficacy between ultrasonographic radiomics models and CECT. Both of the these methods have high diagnostic efficiency and there was no significant difference between the comprehensive model and CECT. Moreover, an external validation cohort was used to assess the diagnostic performance of different models. The AUC of the comprehensive model was 0.90 in the external validation cohort, demonstrating a good predictive ability and robustness on new data. Thus, the comprehensive model based on ultrasonographic radiomics and clinical ultrasound imaging features could provide a convenient, inexpensive, and radiation-free examination for small ccRCC patients.

In this study, we applied a “multivariate filtering” feature selection method, the MRMR algorithm, to maximize the correlation between the imaging features and the prediction target as far as possible. Meanwhile, the correlation between the individual features was minimized as far as possible, with the help of high computational speed and high discriminative power. Features were selected from multiple perspectives to minimize information loss in our study, thus avoiding overfitting or underfitting of the predictive model. Moreover, we used the same ratio to divide the training and validation cohort in both ccRCC and RAML, to ensure the stability of the prediction results. Finally, we chose random forests to build the model classifiers to ensure high overfitting resistance and stability.

There are several limitations in this study. Firstly, the cases in this retrospective study are only from three medical institutions, and the results of the study may be subject to selection bias. Secondly, the ultrasound and CECT images in this study are from different diagnostic apparatuses, and there may be heterogeneity in the study images. In addition, the manual segmentation of outlining the ROI may reduce the reproducibility of this study. In the future, we will verify the stability of the results through multicenter prospective studies.

In conclusion, It is feasible to establish a diagnostic prediction model by ultrasonographic radiomics features in ccRCC and RAML with a maximum diameter of ≤4 cm, and we find that ultrasonographic radiomics features have great potential in identifying tumor heterogeneity in these patients. The comprehensive model showed a superior diagnostic ability in identifying ccRCC, which was similar to that of CECT, providing valuable information for clinicians to make personalized treatment decisions.

## Data availability statement

The raw data supporting the conclusions of this article will be made available by the authors, without undue reservation.

## Ethics statement

The studies involving humans were approved by Ethics Committee of Tianjin Medical University Cancer Institute and Hospital. The studies were conducted in accordance with the local legislation and institutional requirements. The participants provided their written informed consent to participate in this study. Written informed consent was obtained from the individual(s) for the publication of any potentially identifiable images or data included in this article.

## Author contributions

FY: Writing – original draft, Conceptualization. DZ: Data curation, Writing – original draft. L-HZ: Data curation, Writing – original draft. Y-RM: Formal analysis, Writing – original draft. JM: Methodology, Writing – original draft. H-LW: Resources, Writing – review & editing. XW: Validation, Conceptualization, Writing – review & editing. C-WL: Conceptualization, Methodology, Visualization, Writing – review & editing. LP: Data curation. S-QY: Data curation.
